# Fatigue in Cancer Patients in Palliative Care—A Review on Pharmacological Interventions

**DOI:** 10.3390/cancers13050985

**Published:** 2021-02-26

**Authors:** Caritha Klasson, Maria Helde Frankling, Carina Lundh Hagelin, Linda Björkhem-Bergman

**Affiliations:** 1 Department of Neurobiology, Care Sciences and Society (NVS), Division of Clinical Geriatrics, Karolinska Institutet, Blickagången 16, Neo floor 7, SE-141 83 Huddinge, Sweden; caritha.klasson@ki.se (C.K.); maria.helde.frankling@ki.se (M.H.F.); 2 Palliative Medicine, Stockholms Sjukhem Foundation, Mariebergsgatan 22, SE-112 19 Stockholm, Sweden; 3 Department of Health Care Sciences, Ersta Sköndal Bräcke University College, Palliative Research Centre, P.O. Box 11189, SE-100 61 Stockholm, Sweden; carina.lundh-hagelin@esh.se; 4 Department of Neurobiology, Care Sciences and Society (NVS), Division of Nursing, Karolinska Institutet, Alfred Nobels Alle 23, SE-141 83 Huddinge, Sweden

**Keywords:** fatigue, palliative care, cancer, cancer related fatigue, clinical trials, therapeutics

## Abstract

**Simple Summary:**

Cancer related fatigue is a common and distressing symptom for patients with cancer during and after primary treatment, and also in the palliative phase of the disease trajectory. This review focuses on the pharmacological treatment of cancer related fatigue in patients with advanced or metastatic cancer. There are few high-quality studies performed in this setting, but both methylphenidate and corticosteroids might be used to relieve fatigue.

**Abstract:**

Fatigue is one of the most distressing symptoms experienced by cancer patients. The suggested biological mechanism for cancer related fatigue (CRF) includes immune activation triggered by tumor tissue or by anticancer treatment but other mechanisms have also been proposed. Previous large meta-analysis of interventions on fatigue focuses mostly on patients early in the disease trajectory, with only one tenth of included studies performed in palliative cohorts. The aim of this narrative review is therefore to present a background on CRF with focus on the palliative setting. A summary of recent randomized, controlled trials on pharmacological interventions on CRF in palliative care is presented, including studies on psychostimulants, corticosteroids, testosterone and melatonin. Interestingly, in several of these studies there was a positive and similar effect on fatigue in both the intervention and the placebo arm—indicating an important placebo effect for any pharmacological treatment. In addition, studies on dietary supplements and on pharmacological complementary medicines are discussed. To conclude, the evidence is still weak for using pharmacological treatments on CRF in palliative care patients—although methylphenidate and corticosteroids might be considered.

## 1. Introduction

### 1.1. Cancer Related Fatigue

Fatigue is one of the most distressing symptoms experienced by patients with cancer and has a high prevalence in cancer patients [1]. Fatigue can occur before, during and after treatment and persist for a long time [2,3]. Different definitions have been used, but today the most widely used definition stems from the National Comprehensive Cancer Network, NCCN, “Cancer-Related Fatigue is a distressing, persistent, subjective sense of physical, emotional, and/or cognitive tiredness or exhaustion related to cancer or cancer treatment that is not proportional to recent activity and interferes with usual functioning” [4].

The concept of fatigue as a multidimensional symptom has been challenged by the notion that fatigue rather should be regarded as a set of multiple symptoms to be addressed separately [5,6]. The trajectory of fatigue may also change in accordance with development of the cancer disease, with physical but not mental fatigue reported to be more severe in advanced stage cancer patients compared to cancer survivors or healthy individuals [7,8,9]. Cancer related fatigue (CRF) could have a negative impact on patients physical, psychological, social and existential wellbeing, with an impaired experience of quality of life (QoL) as a result [3]. Most research regarding treatment strategies to reduce CRF has been performed in younger patients, with a bias towards women with breast cancer during or after primary treatment [10].

Various measurements are being used to assess presence, frequency and severity of symptoms in palliative care [7,11,12]. The presence of fatigue in patients with cancer with palliative needs is of risk of being overseen by health care personnel due to difficulties in identifying the symptom but also due to a lack of enhanced knowledge of mechanisms causing fatigue. There is a risk that patients suffering from fatigue underreport the symptom if assessment is not made or if health care personnel do not acknowledge the symptom and bring it up for discussion [13]. Patient reported outcome might differ from physician reported outcome [14], suggesting the importance of using validated assessment tools when measuring fatigue.

### 1.2. Cancer Related Fatigue in Patients in Palliative Care

The World Health Organization (WHO) stresses the importance of alleviating distressing symptoms to improve QoL in patients suffering from a life shortening disease [15]. Early identification and symptom management has a key role in palliative care regarding the patients’ physical, psychosocial and existential needs. The improvement of treatments of non-curable diseases can facilitate a prolonged lifetime for patients with life shortening diseases, hence the importance of an active palliative care to improve QoL [16]. The recently proposed revision of the definition of palliative care emphasizes the need for evidence-based practice [17].

Continuous improvement of oncological treatment strategies has steadily improved survival times for patients with cancer in the palliative phase. During these longer disease trajectories, fatigue is a clinical problem for many individuals. Aggregated data from studies on cancer patients with fatigue as a primary outcome show that physical activity and psychological interventions are the best method to alleviate symptoms [10]. However, increased physical activity may not always be an option in the palliative phase, and pharmacological methods must therefore be considered. Neither have psychological interventions been shown to relieve fatigue in patients with advanced stage cancer during and after primary treatment [18], in contrast to patients earlier in their disease trajectory [10]. There is a lack of studies on both physical activity and psychological interventions on patients in the late palliative phase of the cancer disease.

In a previous study on fatigue in patients admitted to palliative care (*n* = 228) it was shown that fatigue increased closer to death [8]. However, fatigue was more distressing and associated with impaired QoL in patients with 2–6 months left in life while the association between fatigue and QoL disappeared during the last days and weeks of life [8].

### 1.3. Previous Reviews and Meta-Analyses of Cancer Related Fatigue, Aim of the Present Review

Since data on interventions regarding CRF have been gathered in a variety of different settings using different assessment tools, there has been a large interest in aggregating data in reviews and meta-analyses of interventions, to be able to draw firmer conclusions regarding best practice. In systematic reviews targeting all aspects of CRF, no subgroup analysis of palliative cohorts has been performed. Reviews focused on data on palliative patients have also included patients with non-cancer diseases [10,19,20], were published some years ago and do therefore not include results from recently published randomized controlled trials (RCTs) [1,21], or focus solely on one type of pharmacological treatment [22]. No review has yet evaluated data specifically on pharmacological treatment of fatigue in palliative cancer patients. The scope of this narrative review is therefore to present a background on CRF with focus on the palliative setting, followed by a thorough review of the literature on presently used pharmacological agents and a discussion of best practice and need for further research. An overview of published reviews in the field from 2014 to 2020 are presented in Table 1.

### 1.4. Epidemiology of Cancer Related Fatigue with Focus on Patients in Palliative Care

Two systematic reviews with meta-analyses on the prevalence of fatigue in cancer patients (all stages) have recently been published [12,23,24,25], with frequency of fatigue in the pooled data reported to be 52 and 49% respectively. A comparison of these two meta-analyses is presented in Table 2. Neither study performed a subgroup analysis of cancer patients in the palliative phase. However, in the study by Ma et al., lower performance status was assessed as the most prominent risk factor for CRF [25]. Similarly, Al Maqbali et al. noted the highest frequency of CRF in patients with advanced disease [12]. In a nationwide Australian study on routinely collected data from more than 116,000 subjects, 80% of cancer patients reported fatigue during the last 60 days of life, and 50% assessed fatigue level to be moderate or severe [26].

### 1.5. Etiology of Cancer Related Fatigue

**Figure 1:**
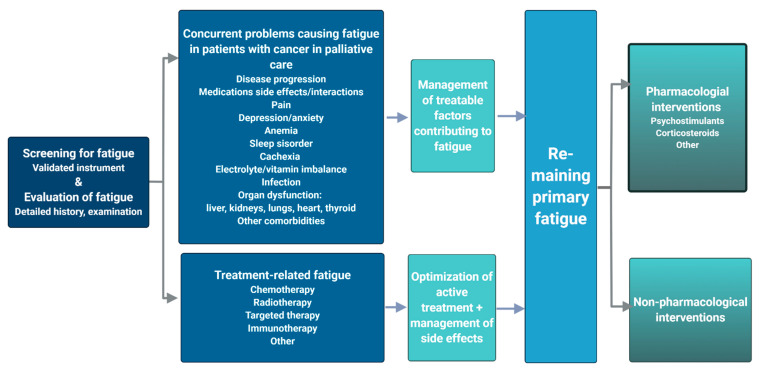
Etiology and management of cancer related fatigue. Made with Biorender.com. Adopted from similar figures in previous work [4,13,23,24].

Fatigue in cancer patients in the palliative phase may be due to both preventable and treatable concurrent conditions, e.g., depression, infections, anemia, sleep disorders, pain, vitamin imbalance, etc. (Figure 1). These should be identified through a detailed medical history, physical examination and relevant investigations [4] and treated according to guidelines/best practice with regard to patients’ wishes and functional status [24]. Additionally, side effects of active oncological treatment include fatigue, and it is important to screen patients in a palliative phase of their disease trajectory for self-assessed fatigue to optimize systemic oncological treatment. Further, fatigue is as such a self-assessed, subjective measure subject to variations in social, emotional and existential status [3,27].

The mechanistic, pathophysiologic underpinning for cancer related fatigue that remains when other factors have been treated/excluded, so called “primary fatigue” according to European Association of Palliative Care (EAPC) 2008 [2], is elusive, but several underlying mechanisms have been proposed in recent reviews [3,28,29]: peripheral immune activation and inflammatory dysfunction due to both to immune system response to tumor tissue and triggered by anticancer treatment; skeletal muscle and mitochondrial dysfunction (in conjunction with cancer cachexia) and neuronal disorders. This adheres well to the categorization of causative concepts by Bower et al. in 2014 in two categories—inflammation and neuroendocrine alterations.

The etiology of fatigue may differ in the early palliative phase compared to the late phase. In the early phase, fatigue and change in fatigue often show an association with inflammation biomarkers [30]. However, in the late stage, close to death, an improvement in fatigue is often difficult to achieve and recovery of inflammatory biomarkers is seldom possible. The experience of fatigue in the very last days of life is also influenced by other symptoms, and the fact that life comes to an end. Despite the same influential etiology, the experience and meaning of fatigue can differ in End-of-Life compared to earlier palliative phases [8].

Both the European Society for Medical Oncology, ESMO and the United States National Comprehensive Cancer Network (NCCN), and other national medical organizations publish consensus-based clinical practice guidelines regarding management of cancer related fatigue with recent updates available [4,31,32]. ESMO does not differentiate between patients in different phases of their disease in their guidelines, but considers patients aged 65 or above separately, since little data on both fatigue assessment and best treatment practice is available for this group [31]. NCCN identifies three groups of cancer patients: undergoing active treatment with focus on curative treatment, post treatment patients and patients in End-of-Life, but does not specifically consider patients in an earlier palliative phase [4]. According to NCCN guidelines, both methylphenidate and shorter courses of corticosteroids can be considered in End-of-Life management of CRF. ESMO, on the other hand, could not reach a consensus on the benefits regarding methylphenidate, but advocates shorter courses of corticosteroids in patients with metastatic cancer [31]. No other pharmacological interventions are deemed beneficial in this setting by either ESMO or NCCN.

## 2. Materials and Methods

This review is conducted as a narrative review of pharmacological interventions on CRF in palliative care to present an overview of different studies being performed up until the date November 2020. Searches have been performed in Medline with the following term both as MeSH term and as free text: fatigue, neoplasm, therapeutics, cancer related fatigue, palliative care, randomized controlled trials and treatment outcome. The terms have been combined in different search blocks of three or more terms combined. More than 500 titles have been screened. In addition, the systematic reviews and meta-analysis mentioned above have been reviewed to find eligible studies [1,10,12,19,20,21,25]. The inclusion criteria for studies presented in Table 3 were randomized, controlled trials, patients ≥18 years with any type of advanced cancer and/or in a palliative care setting with a pharmacological intervention with a control group and with outcome measure of effect on CRF.

## 3. Results

In Table 3 all randomized controlled trials on pharmacological interventions fulfilling the inclusion criteria are presented. In total, 17 studies were included in this review with a total of 1296 patients participating in the trials. The primary objective of the studies was improvement in fatigue, CRF and treatment induced fatigue in patients with cancer. Patient reported outcomes were measured with a variety of different assessment tools.

In a separate section randomized controlled studies on dietary supplements and pharmacological complementary and alternative medicine (CAM) are presented. This section includes studies performed also in non-palliative cancer patients due to the lack of palliative care studies—in contrast to the studies presented in Table 3 where only palliative care cohorts are selected.

### 3.1. Modafinil

Several trials of the use of psychostimulant and effect on CRF have been conducted but show conflicting or weak evidence. In one randomized study of 208 patients with advanced lung cancer receiving modafinil or placebo for 28 days showed improved fatigue in both treatment arms and no difference between placebo and intervention [41]. Additionally, in a randomized placebo-controlled study of 81 patients with glioma receiving radiotherapy, armodafinil had no significant effect on fatigue [40]. A lack of significant effect on fatigue was also presented in a placebo controlled RCT in patients (*n* = 81) with advanced prostate and breast cancer receiving 200 mg Modafinil a day during chemotherapy [42].

### 3.2. Metylphenidate and Dexamphetamine

In contrast, a placebo controlled RCT of men with prostate cancer treated with luteinizing hormone releasing hormone (LHRH) agonist (*n* = 24) revealed a significant improvement with methylphenidate 5–10 mg/day for 10 weeks [35]. Additionally, a RCT of patients with advanced cancer (*n* = 38) showed a significant effect of methylphenidate 10 mg two hours after intake compared to the placebo [34]. However, a dose of 20 mg dexamphetamine for 8 days could not establish significant effect on fatigue in a study of patients with advanced cancer (*n* = 50), even if fatigue was significantly improved on day 2 [43]. Yet another study of patients with breast cancer (*n* = 42) receiving methylphenidate 18 mg/day for two weeks could not show a significant effect over the placebo [37]. Additionally, no significant effect was evident in a placebo controlled randomized crossover study (*n* = 43) of patients with advanced cancer receiving methylphenidate 10 mg for 3 days [36]. In contrast, improved fatigue was detected in men with advanced prostate cancer receiving methylphenidate for 6 weeks compared to the placebo group [39]. However, the study was underpowered since the intervention group only had 10 completers [39]. A mixed 4-arm placebo-controlled intervention (*n* = 140) of methylphenidate 5–20 mg/day and nursing telephone intervention established an improvement in fatigue on day 8 but not at the end of trial at day 15. Nor methylphenidate or nursing telephone intervention showed a statistically significant effect in improved fatigue compared to placebo even if overall symptom burden was decreased [38]. Additionally, a recent placebo controlled RCT (*n* = 100) could not establish evidence for the use of methylphenidate 10 mg/day for 6 days, even if fatigue was significantly improved in both groups [33].

### 3.3. Corticosteroids

Corticosteroids have in randomized trials showed to alleviate CRF. Patients with advanced colorectal cancer showed improved fatigue, receiving dexamethasone 2 mg/day for four weeks during chemotherapy in a randomized placebo-controlled study [14]. In addition, dexamethasone 8 mg/day for two weeks have also provided significant effect in alleviating fatigue in patients with advanced cancer (*n* = 84), in a randomized placebo-controlled trial [48]. In contrast, a randomized placebo-controlled study of patients with advanced cancer receiving methylprednisolone 32 mg/day for 7 days also showed no effect [47]. Additionally, this study was powered for 110 patients but succeeded only to include 57 participants for the analysis. However, a significant effect in alleviating fatigue was established in a placebo-controlled RCT of patients with advanced cancer (*n* = 49) receiving methylprednisolone 32 mg/day for one week [46].

### 3.4. Melatonin

Studies have been performed to test if improved sleep quality could alleviate cancer related fatigue. In a randomized placebo-controlled crossover study, patients with advanced cancer received melatonin 20 mg/day or placebo for one week, which had no significant effect on improving fatigue [44].

### 3.5. Testosterone

Evidence for an effect in testosterone treatment in fatigue is yet not established due to few RCT’s performed. A randomized placebo-controlled study of men with low testosterone and advanced cancer (*n* = 29) could not provide significant evidence for the use of testosterone 150–200 mg for alleviate fatigue after 29 days of treatment [45]. However, at day 72, a significant effect was seen in the intervention arm in one of the subscale scores.

## 4. Dietary Supplements and Complementary Medicine

Different dietary supplements and pharmacological complementary and alternative medicines (CAMs) have been suggested to reduce CRF. However, there are only a few randomized studies performed in the palliative setting.

### 4.1. Palliative Cancer Care Studies

There are two randomized, placebo-controlled, double-blind studies performed in patients with advanced cancer, comparing L-carnitine with placebo [49,50]. Carnitine is an amino acid involved in the energy metabolism within the cell and it is suggested that a deficiency may contribute to fatigue. The first pilot study included 29 patients with advanced cancer and carnitine deficiency at baseline and the larger study included 376 patients with invasive malignancy. Both studies showed no effect of the intervention.

Ginseng has been used for several different conditions in the traditional Chinese medicine for thousands of years. Only one randomized, placebo-controlled, double-blind study using American Ginseng (Panax ginseng) on the treatment of CRF has been performed in patients with advanced cancer (*n* = 112) [51]. Interestingly, an improvement of fatigue was shown in both treatment arms, but ginseng was not superior to the placebo.

It has been suggested that correction of vitamin D deficiency may have a positive effect on fatigue in palliative cancer patients [52,53,54]. This is currently investigated in a large ongoing randomized, placebo-controlled, double-blind study, “Palliative-D” [55]. The results from this study are still not published but in the baseline data from the study cohort (*n* = 530) there was a significant association between low vitamin D levels and severe fatigue in men but not in women [56].

### 4.2. Non-Palliative Cancer Care Studies

Except for the above-mentioned studies, the majority of studies on CAM have been performed in cancer patients in an earlier, non-palliative phase, of the disease. These studies include two randomized studies on the antioxidant Q10 that showed no or limited effect [57,58] and four negative RCTs on the herbal drug Guarana [59,60,61,62]. In contrast, Tualang honey, suggested to have anti-inflammatory and antioxidative properties, was reported to have a significant positive effect on both fatigue and QoL compared to vitamin C treatment in a randomized, open-label study on head-and-neck cancer patients [63].

In contrast to the ginseng study in advanced cancer patients mentioned above, ginseng has shown promising effects in studies performed in cancer survivors and cancer patients in an earlier stage of the disease [64,65,66]. One of these studies was a large multicenter, double-blind RCT (*n* = 364) showing that American ginseng was superior compared to placebo after 8 weeks treatment [64]. All three studies report that the treatment was safe and well tolerated with no serious adverse events.

In a recent meta-analysis of traditional Chinese medicine (TCM) for treatment of CRF 17 randomized controlled trials of different herbal therapies were identified, of which the majority showed positive effects [67]. None of these studies were performed in a palliative care setting. The authors conclude that several of the herbal studies had poor methodological quality, with especially a lack of adequate randomization procedure and blinding.

## 5. Discussion

The result from this review showed that methylphenidate and corticosteroids may improve CRF in the palliative phase [14,34,35,39,46,48] but there is not enough evidence to propose use of modafinil, dexamphetamine, melatonin or testosterone in this setting. It should be noted that the studied cohorts are heterogeneous and include patients both in the early and late palliative phase. Since fatigue is experienced differently throughout the disease trajectory and seems to affect QoL more in the early than in the late palliative phase [8], the comparison of results from these heterogeneous studies are problematic. Thus, the recommendations for different pharmacological agents to relieve fatigue are difficult.

The expert panel from NCCN focuses on patients during/after primary treatment and End-of-Life patients and does not specifically offer guidance on fatigue treatment in patients receiving active oncological treatment in a palliative setting [4]. Patients with metastatic disease receiving active oncological treatment may have a life expectancy of several years, and results from these cohorts cannot readily be compared with results from studies in patients with a life expectancy of a few weeks. Meta-analyses and group comparisons are therefore of little use at present, and instead we suggest that each study should be evaluated individually regarding generalizability of results. Unfortunately, characterization of disease severity in patients included in several studies in this review is scarce, and survival time is only accounted for in two trials [14,33]. Instead, inclusion and exclusion criteria, description of care facilities from which patients were recruited, assessed performance status at baseline and data on dropout rates and reasons for not completing intervention can be used to estimate general disease burden in studied cohorts. Additionally, this is discussed more in detail in the following sections.

### 5.1. Psychostimulants (Methylphenidate, Dexamphetamine and Modafinile)

Psychostimulants was the most commonly studied pharmacological interventions in studies on CRF in palliative care [33,34,35,36,37,38,39,40,41,42,43]. The results in this review revealed a significant effect on the primary outcome only in three trials out of eleven on methylphenidate and modafinil [34,35,39]. Several trials indicated partial, or a trend towards a positive effect during the time of intervention. Interestingly, several studies showed a significant effect in both treatment arms with similar effects in both placebo and intervention groups indicating an important placebo effect for any pharmacological treatment [33,36,37,38,39,40,41,42,43]. Most of the included studies were underpowered resulting in inconclusive data [33,35,37,38,39]. Regarding methylphenidate, earlier studies indicate that they can reverse sedating effects of opioids [68,69] and reduce depressive symptoms [70], both contributors to fatigue. However, a more recent study failed to see an association between effect of methylphenidate and daily opioid dose or depression [71].

Regarding RCTs on psychostimulants, we assessed that three studies were conducted on patients late in the disease trajectory: Centeno [33]—patients recruited from palliative care centers with short remaining life expectancy, Pedersen [34]—palliative inpatients with heavy symptom load and Mitchell [36]—patients who were not candidates for active oncological treatment recruited from palliative care facilities. Other trials mix palliative and curative patients recruited from oncology clinics [35,37], or include very little information on disease severity of randomized patients, rendering interpretation of results more difficult [38,39]. The trial on dexamphetamine [43] was also assessed as being performed in late stage palliative patients.

The three studies on modafinil all recruited patients from oncology centers in comparatively good performance status. Lee studied the effect of modafinil in patients with glioma with good physical performance status, starting high-dose radiotherapy and in some cases concomitant radiotherapy + chemotherapy [40]. Spathis et al. [41] recruited both possibly curative patients (lung cancer stage 3a/3b having completed first line treatment) and patients with metastatic and recurrent disease. Still, dropout due to deterioration or death was only 8%, substantially lower than the expected 25–40% in more severely diseased palliative cohorts [41]. Hovey et al. recruited patients with ongoing docetaxel therapy, with no dropout due to deterioration or death during the intervention period, indication a cohort in early palliative phase [42].

Psychostimulants in low doses, as used in the palliative care setting, are often well-tolerated and have no or only mild side-effects reported as evenly distributed between treatment and placebo arm [33,35,36,37,38,39,40,41]. There seems to be responders and non-responders to this treatment [36,43]—and thus it might be suggested to test this treatment option despite the limited evidence from the underpowered studies. The effect has a rapid onset and is often evident already after the first dose. Thus, the effect could be evaluated already after a few days and terminated if there is a lack of effect or continued if the effect is positive. Methylphenidate might be considered as a first choice since it is the only psychostimulant that has shown a superior effect over placebo in three RCTs [34,35,39] while all RCTs on modafinil have been negative [40,41,42].

### 5.2. Corticosteroids

In clinical practice, corticosteroids are widely used in cancer patients in the palliative phase to alleviate inflammatory pain, relieve nausea, reduce peritumoral oedema, increase appetite, reduce itching and to reduce fatigue [72,73]. As shown above, clinical trials investigating the effect of corticosteroids on fatigue in patients with advanced or metastatic cancer are scarce and partly based on observational studies [74,75]. Of the four RCTs included in this review one showed negative results [47]. Thus, treatment decisions are based rather on empirical knowledge rather scientific evidence, and neither optimal dose nor length of treatment has been established. Side effects of corticosteroids in this setting have not been systematically assessed in clinical trials [74,76]. Still, NCCN for the first time now include short courses of corticosteroids as a treatment options for End-of-Life patients [4]. Further trials on the use of corticosteroids in to relieve fatigue in palliative care are warranted. In their review from 2014, Yennurajalingam et al. argue that trials to study pharmacological interventions in fatigue should be tailored to recruit patients that are most likely to experience a beneficial effect of the studied compound [1]. In the case of steroids, they proposed to study the effect in patients with evident inflammation, measured with CRP, cytokines or other agents [1]. Other ideas would be to study the effect and side effects of corticosteroids on fatigue in patients with liver metastases irrespective of CRP-levels, slightly longer treatment periods with lower doses, and the effect of repeated shorter courses (as *n*-of-1-trials). Regarding studies on corticosteroids, three out of four included trials are assessed as including patients in the later phases of their disease trajectories [46,47,48], whilst the study by Tanioka [14] included patients with ongoing active treatment and overall survival of 6.8 months.

### 5.3. Melatonin

The RCT on melatonine [44] had relatively large dropout rates due to deterioration or death, and thus included many end-stage cancer patients. Interestingly, melatonin has, in addition to its effect on sleep disorder, been suggested to have anti-inflammatory and antioxidative effects on a cellular level [77]. Thus, we believe that melatonin is an interesting agent for treatment of fatigue and deserves further studies.

### 5.4. Testosterone

The Del Fabbros trial on testosterone involved oncological outpatients with metastatic or recurrent cancers, with no dropout during follow-up, indicating that this cohort had a comparatively long life expectancy [45].

### 5.5. Complementary and Alternative Medicine

A recent review suggests the need of addressing CAM in treating CRF since patients may use this as part of self-care, alongside with traditional treatment methods without informing health care providers [78]. Except for three high-quality studies performed in patients with advanced cancer, including interventions with L-carnitin and ginseng [49,50,51], no studies have been performed in the palliative setting. All three studies showed negative results. Indeed, most pharmacological CAM interventions on cancer patients in an earlier phase of the disease are also negative or are have been performed with low methodological quality. An exception might be the high-quality study performed on ginseng that shows promising effects on CRF in cancer survivors [64]. A review of the use of guarana, ginseng, acetyl L-carnitine, Q10 enzyme and green algae demonstrates that trials are mostly performed with women with breast cancer in all stages and does not specifically target palliative care [79]. Further research of CAM in palliative context is needed to provide evidence.

### 5.6. Placebo Effect

Interestingly, several of the studies described in this review show an improvement of fatigue both in the intervention and the placebo arm where the intervention is not superior to placebo. Two recent reviews have highlighted that placebo may affect fatigue in patients with cancer [80,81], which is an important confounding factor when analysis of intervention is performed. The authors to one of these reviews recommend that it might be necessary to develop alternative trial designs to better account for the placebo response when studying effect on fatigue [81]. A study of placebo responses on CRF confirmed that up to 29% of patients with advanced cancer experienced improved fatigue compared to usual care [82], indicating that the mechanism behind responses are complex. However, in the clinical setting also the placebo effect is an important effect. If the treatment has no adverse side effects any positive effect of the treatment is beneficial for the patient.

### 5.7. Mixed Interventions

There is a lack of trials using methods of both pharmacological and non-pharmacological methods to alleviate fatigue. However, studies performed with mixed interventions have also failed to show a significant effect in alleviating fatigue [38]. NCCN recommends primarily non-pharmacological approaches, such as physical activity, counseling and nutritional support to treat CRF after treatment and in End-of-Life care. This is supported in a recent review comparing pharmacological and non-pharmacological interventions on fatigue in patients with cancer during and after treatment [10].

### 5.8. Clinical Applications

According to the findings presented here corticosteroids and methylphenidate might be the drugs of choice for pharmacological treatment of fatigue in palliative cancer care. However, the placebo effect is not negligible for any of the treatments studied. In addition, the patients included in the studied presented here is heterogeneous and patients in the very End-of-Life is mostly missing in clinical trials. Thus, when the optimal treatment for an individual patient is decided, several factors have to be taken into account, such as the performance status of the patient, the motivation and belief in the suggested treatment, and which adverse reactions that should be avoided to sustain the QoL of this specific patient. Above all, the treatment should not harm the patient in any way. Thus, we recommend an individualized treatment approach rather than a generalized recommendation that fits all.

## 6. Limitations and Strengths

This review had several limitations. First, this is a narrative review and no meta-analysis was performed. Second, only RCT trials were included, targeting the palliative phase, and there may be some trials left out, which may result in a lack of efficiency detected in other pharmacological interventions or with different design. Third, trials combining non-pharmacological and pharmacological interventions have not been included. Additionally, the variety of assessment tools makes it difficult to identify the impact of coexisting factors affecting the effect on CRF such as the psychological, physical and emotional status and depression. A strength of this review is the focus on CRF and pharmacological interventions in cancer disease in the palliative phase, which may differ from the pharmacological effects in curable disease.

## 7. Conclusions

To conclude, the evidence is still weak for using pharmacological treatments on fatigue in palliative care patients, although methylphenidate and corticosteroids might be considered. Further research is needed regarding pharmacological treatments on CRF in patients with advanced cancer admitted to palliative care, and in combination with non-pharmacological interventions, and about CRF in End-of-Life. Additionally, further research of biological mechanisms affecting fatigue is needed to support and raise new research questions and interventions.

## Figures and Tables

**Table 1:** Reviews on cancer related fatigue.

Review	Mücke [19]	Yennurajalingam [1]	Qu [21]	Mustian [10]	Tomlinson [20]
Time, until	April 2014	June 2014	July 2014	May 2016	May 2017
Type	Systematic review, MA	Narrative review	Systematic review, MA	Systematic review, MA	Systematic review, MA
Inclusion criteria	RCTs of adults in palliative care (cancer, non-cancer)	RCTs of adults in palliative care (cancer, non-cancer)	RCTs of adults with cancer	RCTs of adults with cancer	RCTs and quasi-RCTs, patients with cancer or recipients of HSCT
Cancer stage	Advanced /metastatic	All stages	All stages	All stages	All stages
Objective	To compare effect of pharmacological treatment of fatigue to control interventions	To review pharmacological interventions for CRF.	To examine the effect and safety of Methylphenidate and Modafinil in treatment of CRF	To compare effect of exercise, psychological, a combination thereof, or pharmacological interventions	To compare effect of pharmacological treatment of fatigue to control interventions
Outcome	Fatigue severity + change	Fatigue severity (CRF)	Fatigue severity (CRF)	Fatigue severity (CRF)	Fatigue severity + change
Included studies/subjects	45/4696	18/2855	10/1582	113/11 525	117/19 819
Palliative studies (cancer)	40%	Not reported (narrative)	Not reported	10% metastatic, 30% mixed	17%
Conclusions regarding pharmacological interventions	Methylphenidate may be advantageous. Too little evidence for corticosteroids. Not enough evidence to support use of specific drug.	Adheres to guidelines (2014). Recommends future research with more personalized interventions.	Methylphenidate but not Modafinil reduced CRF and may be beneficial for the treatment of CRF.	Exercise, psychological intervention + their combination reduces CRF. As a group, pharmaceutical interventions are not effective during and after cancer treatment.	As a group, stimulants were not effective. Methylphenidate improved fatigue, while Modafinil and corticosteroids did not.

Abbreviations: MA: meta-analysis, RCT: randomized controlled trial, CRF: cancer related fatigue.

**Table 2:** Comparison of two meta-analyses on the prevalence of cancer related fatigue.

Study	Ma et al. [25]	Al Maqbali et al. [12]
Inclusion/exclusion criteria	Observational studies (>50 participants) on patients with cancer. Included studies reported diagnostic criteria for CRF and prevalence of CRF or risk factors of CRF.	Cross sectional or baseline data from cohort studies (>50 participants), patients with cancer aged >15. Fatigue measured on multi-item scales.
Number of screened/ included articles	2641/84	10,516/129
Number of subjects	144,813 (31% male)	71,568 (51% male)
Cancer related fatigue (%)	52 (95% CI 48–56)	49 (95% CI 45–53)
*Subgroup analysis*	*Risk factors for fatigue* *(OR, 95% CI)*	*Frequency* *(%)*
Strongest association	Poor performance status (6.58, 2.60–16.67)	Ongoing treatment (62)
	Insomnia (2.83, 1.22–6.57)	Advanced stage (61)
	Pain (2.64, 1.20–5.80)	Mixed cancers in study (57)
	Chemoradiotherapy (2.25, 1.90–2.67)	
	Depression (2.23, 1.70–2.92)	
	Female sex (2.07, 1.51–2.84)	
Weakest association	Neuroticism (1.23, 1.05–1.43)	
	No statistical significance: Low income, comorbidities, anxiety, physical exercise	
Separate analysis of patients in palliative phase	No	No

Abbreviations. CRF: cancer related fatigue, CI: confidence interval, OR: odds ratio.

**Table 3:** Summary of randomized controlled trials on pharmacological treatments of fatigue in patients with advanced cancer.

Study	Population	Study Design	Intervention	Comparative Intervention	Primary Outcome (Assessment Tool)	Comments
**Methylphenidate (MPH)**						
Centeno 2020 [33] Spain Advanced cancer	ITT = 100, PP = 77 Intervention: Mean age = 66 Men = 52% Placebo: Mean age = 68 Men = 53%	Randomized, double blind, placebo controlled	Methylphenidate 10–25 mg/day for 6 days	Placebo	Effect on fatigue after 6 days. (ESAS, FACT-F).	No significant difference between treatment arms (ESAS *p* = 0.52, FACT-F *p* = 0.3). Mean improvement in MPH group: ESAS −2.3 (SD 2.6), FACT-F −3.4 (SD 2.5) Placebo group: ESAS −1.9 (SD 2.5), FACT-F −2.4 (SD 2.9)
Pedersen 2020 [34] Denmark Cancer, palliative care.	ITT = 38, PP = 28 Intervention: Mean age: 69 Men: 29% Placebo: -	Randomized, double blind, placebo controlled	10 tablets of Methylphenidate 10 mg and 10 tablets placebo, randomly packed	Placebo, own control.	Effect on fatigue after 2 and 5 h. (VAS tiredness).	Significant effect with MPH but not placebo after 2 h (mean difference in decrease -12, SD 20, *p* = 0.004) and after 5 h (mean difference in decrease -12, SD 19, *p* = 0.001)
Richard 2015 [35] Canada Advanced Prostate cancer	ITT = 24, PP = 23 Intervention: Median age = 63 Men = 100% Placebo: Median age = 74 Men = 100%	Randomized, double blind, placebo controlled	Methylphenidate 5–10 mg/day for 12 weeks	Placebo	Effect on fatigue after 10 weeks (FACT-F).	After 10 weeks mean difference in change from baseline was 5.6 points in favor of intervention (95% CI 1.0–10.3), *p* = 0.022.
Mitchell 2015 [36] Australia Advanced cancer	ITT = 43, PP = 24 Intervention: Median age = 71 Men = 52% Placebo: -	Randomized, N-of-1, double blind, placebo controlled crossover, multicycle design.	Methylphenidate 5 mg × 2 for 3 days, placebo for 3 days, methylphenidate 5 mg × 2 for 3 days. 3 cycles.	Placebo for 3 days, Methylphenidate for 3 days, placebo for 3 days. 3 cycles.	Effect on fatigue as individual comparison + population estimate (FACIT-F).	No difference was detected between groups characterized as responders and non-responders after 84 completed cycles, mean difference 3.2 (95% credible interval −2.0, 9.0). 7 patients had clinically significant positive effect of MPH.
Escalante 2014 [37] USA Breast cancer (local/ metastatic)	ITT = 42, PP = 33 Intervention: Mean age = 57 Men = 0% Placebo: -	Randomized, placebo controlled, crossover	Methylphenidate 18 mg/day for 14 days + placebo for 14 days.	Placebo for 14 days + methylphenidate 18 mg/day for 14 days.	Effect on fatigue assessed as improvement of worst level of fatigue after 14 days. (BFI)	No significant difference between treatment groups (*p* = 0.54) regarding worst level of fatigue after 14 days of treatment.
Bruera 2013 [38] USA Advanced cancer	ITT = 190, PP = 140 Intervention: Mean age = 58 Men = 33% Placebo: -	Randomized, 4-arm, placebo controlled	Methylphenidate 5–20 mg/day + nurse telephone intervention OR control telephone intervention for 15 days	Placebo + nurse telephone intervention OR control telephone intervention for 15 days	Effect on fatigue after 15 days (FACIT-F)	All groups showed significant effect in improved fatigue on day 15. MPH was not superior to placebo from baseline to end of trial (5.5 vs. 6.0, *p* = 0.69).
Roth 2010 [39] USA Advanced prostate cancer	ITT = 32, PP = 23 Intervention: Mean age = 68 Men = 100% Placebo: Mean age = 71 Men = 100%	Randomized, double blind, placebo controlled	Methylphenidate 5–30 mg for 6 weeks. Individual titration of dose after day 3.	Placebo	Effect on fatigue after 6 weeks. (BFI).	Significant effect of both MPH and placebo (improvement in BFI total score 3.63, *p* = 0.01 and 2.58, *p* = 0.02), comparison between groups not shown. Methylphenidate reduced BFI severity score more than placebo (*p* = 0.03). RR for fatigue improvement in MPH group was 3.04 (CI 1.04–8.86) compared to placebo (*p* = 0.02)
**Modafinil**						
Lee 2016 [40] USA Glioma	ITT = 81, PP = 62 Intervention: Median age = 56 Men = 57% Placebo: Median age = 54 Men = 53%	Randomized, placebo controlled, multicenter pilot study	Armodafinil 150 mg 8 weeks during radiotherapy. Intervention start within 10 days of RT start.	Placebo	Effect on fatigue after 42 days (FACIT-F).	No significant difference in median change in FACIT-F was detected between armodafinil, −1 (range −22 to 48) and placebo, −3 (range −38 to 22), *p* = 0.30.
Spathis 2014 [41] UK Advanced lung cancer	ITT = 208, PP =160 Intervention: Median age = 68 Men = 49% Placebo: Median age = 69 men = 50%	Randomized, double blind, placebo controlled	Modafinil 100 mg day 1–14, 200 mg day 15–28	Placebo	Effect on fatigue on day 28 (FACIT-F).	No significant effect between treatment arms. Mean score difference between treatment arms 0.20 (95% CI; −3.56–3.97) based on mean score change in modafinil group 5.29 (95% CI 2.57 to 8.02) and placebo group 5.09 (95% CI 2.54 to 7.65).
Hovey 2014 [42] Australia Metastatic breast or prostate cancer	ITT = 83, PP = 66 Intervention: Mean age = 66 Men = 78% Placebo: Mean age = 68 Men = 78%	Randomized 2:1, double blind, placebo controlled, multicenter study	Day 0 + 21: Chemotherapy Day 3–17: Modafinil 200 mg. Min 2, max 4 cycles	Placebo	Effect on chemotherapy-induced fatigue (cumlative MDASI AUC during first 7 days of TP 1 and 2)	No significant effect between treatment arms (MDASI AUC_3–10_ 35.9 vs 39.6, 95% CI −8.9 to 1.4, *p* = 0.15).
**Dexamphetamine**						
Auret 2009 [43] Australia Advanced cancer	ITT = 50, PP = 39 Intervention: Mean age = 73 Men = 64% Placebo: Mean age = 67 Men = 80%	Randomized, double blind, placebo controlled	Dexamphetamine 10 mg × 2 for 8 days.	Placebo	Effect on fatigue after 8 days (BFI).	No significant difference in effect between groups (*p* = 0.414) at day 8. Mean decrease in BFI 1.08 in intervention group vs. 0.98 in placebo group.
**Melatonin (MLT)**						
Lund Rasmussen [44] 2015 Denmark Advanced cancer	ITT = 72, PP = 44 Intervention: Mean age = 65 Men = 29% Placebo: Mean age = 62 Men = 34%	Randomized, placebo controlled, double blind, crossover	Melatonin 20 mg for 1 week, wash out 2 days, placebo for 1 week.	Placebo for 1 week, wash out 2 days, Melatonin 20 mg for 1 week.	Effect on fatigue during first intervention with MLT for one week (MFI-20)	No significant effect of MLT was detected. Mean difference in change between week with intervention and placebo 2.8 units (SD 25.6, *p* = 0.47).
**Testosterone**						
Del Fabbro 2013 [45] USA Advanced cancer, hypogonadal men	ITT = 43, PP = 29 Intervention: Mean age = 57 Men = 100% Placebo: Mean age = 63 Men = 100%	Randomized, double blind, placebo controlled	Testosterone 150–200 mg, injection day 1, 15, 29, 43, 57.	Placebo	Effect on fatigue at day 29. (FACIT-F).	No significant difference in fatigue scores between intervention (4, SD 8) and placebo (−2, SD 12), *p* = 0.12.
**Corticosteroids**						
Paulsen 2014 [46] Denmark Advanced cancer	ITT = 49, PP = 47 Intervention: Mean age = 62 Men = 50% Placebo: Mean age = 66 Men = 52%	Randomized, double blind, placebo controlled	Methyl- prednisolone 16 mg × 2 for 7 days.	Placebo	Effect on pain intensity after 7 days. Fatigue secondary outcome (EORTC-C30).	Significant improved (*p* = 0.003) fatigue in the intervention arm (−17, CI 95%, −27 to −6) compared to worsened fatigue in the placebo arm (3, CI 95%, −5 to 11).
Tanioka 2018 [14] Japan Metastatic colorectal cancer	ITT = 74, PP = 72 Intervention: Median age = 65 Men = 61% Placebo: Median age n = 68 Men = 63%	Randomized, double blind, placebo controlled.	Dexamethasone 2 mg for 4 weeks, 1 week after end of targeted therapy.	Placebo	Effect on fatigue assessed as incidence of fatigue (CTCAE v.4). Assessment by patients and investigators	Significantly less fatigue grade ≥ 2 according to patients (*p* = 0.03), but not investigators (*p* = 0.69).
Eguchi 2015 [47] Japan Cancer, palliative care	ITT = 35, PP = 34 Intervention: Median age = 71 Men = 61% Placebo: Median age = 68 Men = 62%	Pilot randomized, multicenter, double blind, placebo controlled	Methyl- prednisolone 32 mg for 7 days.	Placebo	Effect on fatigue after 7 days (VAS).	No significant difference between groups (*p* = 0.484). Mean change in intervention arm (−1.56, SD 32.5) compared to placebo (−9.06, SD 27.2).
Yennurajalingam 2013 [48] USA Advanced cancer	ITT = 132, PP = 84 Intervention: Median age = 60 Men = 47% Placebo: -	Randomized, double blind, placebo controlled	Dexamethasone 4 mg × 2 for 14 days	Placebo	Effect on fatigue after 15 days (FACIT-F).	Significant improved in intervention group compared to placebo, *p* = 0.008. Mean change from baseline with dexamethasone was 9, (SD 10.3) and with placebo 3.1 (SD 9.59).

Abbreviations: AUC: Area under Curve, BFI: Brief Fatigue Inventory; CI: Confidence Interval; CTCAE v4: National Cancer Institute’s Common Terminology Criteria for Adverse Events; EORTC-C30: European Organization for Research and Treatment of Cancer-Quality of Life Questionnaire 30; ESAS: Edmonton Symptom Assessment Scale; FACIT-F: Functional Assessment of Chronic Illness Therapy-Fatigue; FACT-F: Functional Assessment of Cancer Therapy-Fatigue; I: Intervention; ITT: Intention To Treat; MDASI: MD Anderson Symptom Inventory; MFI-20: Multidimensional Fatigue Inventory-20; mg: milligram; MLT: Melatonin; MPH: Methylphenidate; PP: Per Protocol; RR: Response Rate; RT: Radio Therapy; SD: Standard Deviation; TP. Treatment Period; VAS: Visual Analogue Scale; vs: versus.
